# Rapid and Accurate Detection of *Chrysomya megacephala* (Diptera: Calliphoridae) Using Recombinase Polymerase Amplification Combined with Lateral Flow Dipstick

**DOI:** 10.3390/insects15121008

**Published:** 2024-12-20

**Authors:** Chengxin Ye, Xuan Tang, Fengqin Yang, Xiangyan Zhang, Yanjie Shang, Yang Xia, Yuanxing Wang, Shaojiang Guo, Lagabaiyila Zha, Yadong Guo, Dan Wen

**Affiliations:** 1 Department of Forensic Science, School of Basic Medical Sciences, Central South University, Changsha 410013, China; 2 Public Security Forensic Center of Haidian, Beijing 100089, China

**Keywords:** forensic entomology, insect species identification, recombinase polymerase amplification, lateral flow dipstick, *Chrysomya megacephala*

## Abstract

Estimating the postmortem interval (PMI) is critical in the field of forensic science, and necrophagous insects play a significant role in this process. *Chrysomya megacephala* (Fabricius) (Diptera: Calliphoridae) is a common necrophagous insect species, making its rapid and accurate identification essential. However, commonly used molecular biology methods, such as DNA barcode, still have some limitations in identifying necrophagous insects as they are often complex, time-consuming, and reliant on laboratory instruments. Therefore, in this study, we have developed an innovative detection system for the rapid and accurate identification of *C. megacephala* based on the Cytochrome b gene using recombinase polymerase amplification (RPA) and lateral flow dipstick (LFD) in combination. The developed RPA-LFD detection system achieved complete amplification in just 15 min at 37 °C with good sensitivity and specificity. Only 7.8 × 10^−4^ ng or more of target DNA fragments were required, and a positive detection rate of 100% was achieved in 18 *C. megacephala* samples from actual cases. In addition, the ability of the developed RPA-LFD detection system in combination with rapid DNA extraction methods to enable on-site detection was preliminarily explored. The results suggested that when the RPA-LFD detection system was combined with the grinding ddH_2_O extraction method (a rapid DNA extraction method), the process from species acquisition to visualization of detection results could be completed in less than 20 min. In conclusion, this innovative RPA-LFD detection system outperforms commonly used molecular biology methods for *C. megacephala* identification in terms of speed, sensitivity and convenience, making it suitable for direct application at crime scenes, promising to provide important assistance in estimating PMI and expanding the impact of forensic entomological evidence.

## Simple Summary

Necrophagous insects on corpses play a crucial role in estimating the postmortem interval (PMI) through their community succession patterns and developmental data. Developing a simple, rapid, and accurate method for identifying insect species has become a key focus in forensic entomology. In this study, we collected insect samples from real crime scenes between 2018 and 2023 and identified *Chrysomya megacephala* (Fabricius) (Diptera: Calliphoridae) as the most frequently encountered species. To facilitate its identification, we developed a detection system using recombinase polymerase amplification (RPA) combined with lateral flow dipstick (LFD) technology. This detection system demonstrated high specificity and sensitivity and can be effectively used with rapid DNA extraction methods.

## 1. Introduction

Estimating postmortem interval (PMI) is a crucial task in the field of forensic science. It aids in narrowing the scope of the investigation, providing valuable clues, and assisting in the reconstruction of the crime scene [1]. Forensic entomology, with its focus on the succession patterns and developmental data of necrophagous insects [2,3,4], is particularly adept at PMI estimation for decomposed corpses [5]. The initial step in PMI estimation using this method is the precise identification of necrophagous insect species [6,7]. *Chrysomya megacephala* (Fabricius) (Diptera: Calliphoridae) is found in both Asia and America [8]. As a common and major early colonizer of decaying carcasses, it is of significant value in PMI estimation [9,10].

Current methods for identifying species of necrophagous insects include a morphological method, molecular biology method, and chemical characterization method [11]. The traditional morphological observation method is widely recognized as the gold standard for species identification, which can achieve rapid identification of necrophagous insect species in the field environment [12,13]. However, there are certain limitations associated with this method, including the strong subjectivity of identification, the requirement for the identifier to possess a certain level of knowledge in entomological classification, and the need for intact insect samples for accurate identification [14]. These factors make it challenging to promote the use of morphological methods in forensic practice at the grassroots level. Sperling et al. (1994) pioneered the use of molecular biology methods for identifying species of necrophagous insects [15]. This method has become increasingly popular due to its simplicity and ability to identify necrophagous insect species at various stages of development [16]. However, conventional molecular biology methods have drawbacks, including time-consuming processes and a reliance on laboratory instruments, which limit their practical application at crime scenes. An emerging class of necrophagous insect species identification methods involves the analysis of chemical signatures in necrophagous insects using Fourier transform infrared spectroscopy (FTIR) and Gas Chromatography–Mass Spectrometry (GC-MS) techniques [17,18]. Despite its potential to provide more information, this method is rarely used in forensic science due to its reliance on laboratory instruments and the lack of a comprehensive database containing relevant chemical characteristics. Therefore, there is an urgent need to develop a simple, rapid, and accurate method for identifying necrophagous insect species in field environments to overcome the limitations of existing methods and enhance the practical value of forensic entomological evidence.

Recombinase polymerase amplification (RPA) is an innovative isothermal nucleic acid amplification technique, initially proposed by Piepenburg et al. (2006) [19]. This technique enables rapid exponential amplification at human body temperature within a brief span of 30 min, eschewing the need for costly equipment [20,21]. To render RPA results directly observable, researchers have advocated for the combination of RPA with lateral flow dipstick (LFD) [22,23,24]. Currently, the RPA-LFD is primarily applied in the field of viral, bacterial and parasitic detection, yielding promising results [24,25,26,27,28,29]. Therefore, in this study, we employed RPA-LFD technology to identify forensic necrophagous fly species.

In this study, samples of necrophagous flies were collected from actual death cases in both Southern and Northern China. The most frequently observed necrophagous fly species at the crime scenes, *C. megacephala*, was chosen as the subject of study. We established a detection system targeting the Cytochrome b gene (*Cytb*) of *C. megacephala* using RPA and LFD technologies. The specificity and sensitivity of this detection system were evaluated, and the effectiveness was also assessed using actual case samples. Moreover, the ability of the detection system in combination with rapid DNA extraction methods to enable on-site detection was preliminarily explored.

## 2. Materials and Methods

### 2.1. Sample Collection and DNA Extraction

We collected samples of necrophagous flies from 45 actual death cases in cities (Changsha and Beijing) across Southern and Northern China between the years 2018 and 2023. The collected insect samples were preliminarily identified and classified based on morphological characteristics. These samples were carefully loaded into centrifuge tubes containing anhydrous ethanol and stored at −20 °C for preservation. The total DNA from each collected sample was extracted using the Steady Pure Universal Genomic DNA Extraction Kit (Accurate Biotechnology, Changsha, China), and the concentration of extracted DNA was quantified using the NanoDrop One Microvolume UV-Vis Spectrophotometer (Thermo Fisher Scientific, Waltham, MA, USA).

According to previously published research, a 658 bp fragment of the mitochondrial cytochrome-oxidase 1 gene (*COI*) was amplified and sequenced using Sanger sequencing technology for species identification of necrophagous flies [30,31,32]. Primer details are presented in Table 1, Polymerase Chain Reaction (PCR) reaction conditions were aligned with the study by Guo et al. (2014) [32]. The sequencing was performed by Beijing Tsingke Biotechnology Company, and the sequencing results were compared using the NCBI Blast tool (https://blast.ncbi.nlm.nih.gov/Blast.cgi, accessed on 6 May 2024). The species data from all 45 actual death cases were analyzed and are detailed in Table 2 and Table 3.

### 2.2. RPA Primers Design and Screening

For primers design, a total of 24 species of necrophagous flies were considered. The *Cytb* sequences of these necrophagous fly species were complete and aligned using MEGA6 software of version 6.06 to identify differential sequences [33]. The RPA primers were designed following the guidelines of the nfo DNA isothermal rapid amplification kit (Genenode, Wuhan, China) and using Primer Premier 5.0 software (Table 4) [34]. The specificity of the designed RPA primers was assessed using Primer-BLAST on the NCBI website.

To confirm the specificity of the designed RPA primers, seven common necrophagous fly species were selected: *C. megacephala*, *Lucilia sericata* (Meigen), *Sarcophaga crassipalpis* (Macquart), *Chrysomya rufifacies* (Macquart), *Calliphora vicina* (Robineau-Desvoidy), *Megaselia scalaris* (Loew) and *Sarcophaga peregrina* (Robineau-Desvoidy). Conventional PCR was performed in a 10 µL reaction mixture, comprising 1 µL of 10 mM forward primer, 1 µL of 10 mM reverse primer, 5 µL of 2×TSINGKE Master Mix (Tsingke, Beijing, China), and 3 µL of a mixture containing ddH_2_O and 2 ng DNA template. The PCR products were then separated by agarose gel electrophoresis.

### 2.3. Probe Design

The probe was designed using Primer Premier 5.0 software [34], ensuring it did not overlap with the recognition sites of the RPA primers, and did not possess palindromic sequences, internal secondary structures, or consecutive repetitive bases. The primers and probe were synthesized by Accurate Biotechnology Company, Changsha, China.

### 2.4. RPA-LFD Detection System

The RPA procedure was executed using the nfo DNA isothermal rapid amplification kit (Genenode, Wuhan, China) in a 10 µL reaction volume, following the manufacturer’s instructions. We added 32.9 µL of A Buffer to the dry powder tube to dissolve the dry powder. The reaction mixture included 6.5 µL of A Buffer dissolved in dry powder, 0.4 µL of forward primer (10 µM), 0.4 µL of reverse primer (10 µM), 0.2 µL of probe (10 µM), 2 µL of DNA template (1 ng/µL), and 0.5 µL of B Buffer. Post-incubation, 5 µL of the amplification product was mixed with 195 µL of diluent (Tris-HCL 0.05 M, pH8.0), and then 100 µL of this mixture was added to the sample pad of the LFD single-use nucleic acid test strips (Genenode, Wuhan, China) for analysis. A sample was deemed positive if both the detection (T) line and the quality control (C) line were visible, negative if only the C line was apparent, and invalid if the C line was absent.

### 2.5. Optimal Reaction Conditions for RPA-LFD Detection System

To determine the optimal conditions for the RPA-LFD detection system, we varied the reaction temperatures (25, 37, and 42 °C) and durations (10, 15, 20, and 25 min) to identify the most favorable temperature for initiating amplification and the most effective amplification duration.

### 2.6. Specificity of RPA-LFD Detection System

The RPA-LFD detection system was used to detect *C. megacephala* and six other common necrophagous fly species from actual cases (*L. sericata*, *S. crassipalpis*, *C. rufifacies*, *C. vicina*, *M. scalaris* and *S. peregrina*). Prior to experimentation, the DNA concentration of all necrophagous fly samples was diluted to 1 ng/µL. The detection system mentioned in Section 2.4 and the optimal reaction conditions mentioned in Section 2.5 (37 °C, 15 min) were followed, and the detection process was repeated three times.

### 2.7. Sensitivity of RPA-LFD Detection System

The RPA-LFD detection system was used to detect various concentrations of synthetic target DNA fragments of *Cytb* of *C. megacephala*, as detailed in Table 5 with detailed concentration settings. The target DNA fragments with precise concentrations were synthesized and provided by the Tsingke company, Beijing, China. The detection system mentioned in Section 2.4 and the optimal reaction conditions mentioned in Section 2.5 (37 °C, 15 min) were followed, and the detection process was repeated twice.

### 2.8. Effectiveness of RPA-LFD Detection System

The effectiveness of the RPA-LFD detection system was evaluated using 18 *C. megacephala* DNA samples obtained from actual cases. These samples were not employed in previous experimental sessions. The detection system mentioned in Section 2.4 and the optimal reaction conditions mentioned in Section 2.5 (37 °C, 15 min) were followed.

### 2.9. RPA-LFD Detection System in Combination with Rapid DNA Extraction Methods

The potential applicability of the RPA-LFD detection system for field testing was explored in combination with three rapid DNA extraction methods: grinding ddH_2_O extraction method, water bath ddH_2_O extraction method, and one-step extraction method, as described in extant studies [35,36,37]. The grinding ddH_2_O extraction method involves grinding 2 mg of insect tissue in a centrifuge tube followed by the addition of 200 μL of ddH_2_O, a process that takes approximately 2 min. The water bath ddH_2_O extraction method involves placing 2 mg of insect tissue into a centrifuge tube containing 200 µL of ddH_2_O, and then heating in a water bath at 80 °C for 10 min. The one-step extraction method, following the protocol of the DNA-EZ Reagents V All-DNA-Fast-Out kit (BBI Solutions, Shanghai, China), involves placing 2 mg of insect tissue into a centrifuge tube containing 50 µL of All-DNA-Fast-Out solution, and then heating in a water bath at 80 °C for 10 min. Post-DNA extraction, all extracts were shaken and diluted to a concentration of 1 ng/µL for application in the RPA-LFD detection system. The detection system mentioned in Section 2.4 and the optimal reaction conditions mentioned in Section 2.5 (37 °C, 15 min) were followed.

## 3. Results

### 3.1. Sample Collection

As shown in Table 2 and Table 3, a total of 24 species of necrophagous flies were identified from the samples collected between 2018 and 2023. Among these, the most frequently observed species were *C. megacephala* and *L. sericata*. The necrophagous fly *C. megacephala* was observed in three or more cases in both southern and northern China, indicating its prevalence and making it the primary focus of this study. Additionally, other necrophagous fly species that occurred frequently (in more than three cases) included *S. crassipalpis* (eight cases), *C. rufifacies* (six cases), *C. vicina* (six cases), *M. scalaris* (five cases) and *S. peregrina* (five cases). These species were important for differential identification from *C. megacephala*. Consequently, a total of seven necrophagous fly species (*C. megacephala*, *L. sericata*, *S. crassipalpis*, *C. rufifacies*, *C. vicina*, *M. scalaris* and *S. peregrina*) were included in this study.

### 3.2. RPA Primers Screening

In this study, three pairs of *Cytb*-specific RPA primers were designed to identify *C. megacephala*. These primer pairs were named CM-RPA-1-F/R, CM-RPA-2-F/R, and CM-RPA-3-F/R, as presented in Table 4. As shown in Figure S1, the amplification product results of CM-RPA-2-F/R, when compared to CM-RPA-1-F/R and CM-RPA-3-F/R, displayed clear and bright bands without non-specific amplification. Based on these findings, the primer pair CM-RPA-2-F/R was selected as the optimal primer pair for subsequent experiments. Then, a specific probe with a length of 48 bp was obtained. The probe was modified with a FAM fluorescent group at the 5′ end and a C3-spacer at the 3′ end, and a base 17 bp away from the 3′ end of this probe was replaced with THF (Table 4).

### 3.3. Optimal Reaction Conditions for RPA-LFD Detection System

**Figure 1:**
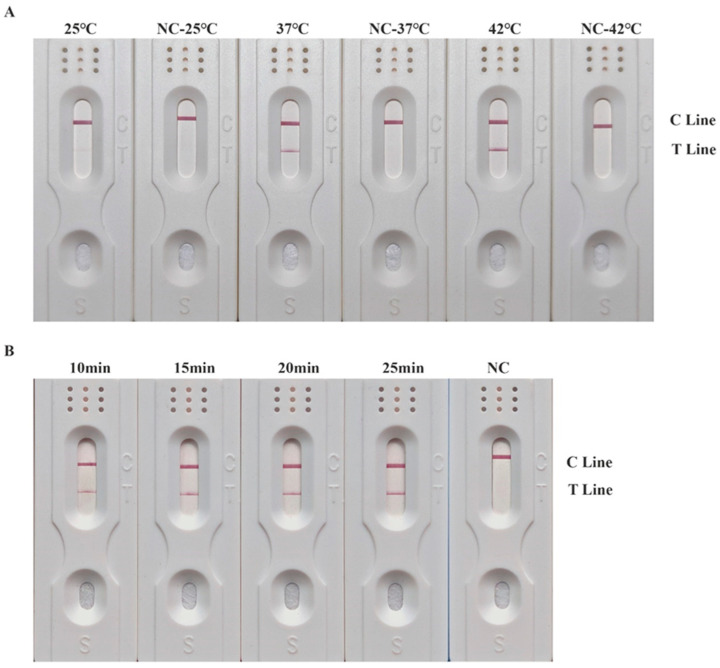
Results of RPA-LFD detection system at different reaction temperatures and reaction times. (**A**) Results of RPA-LFD detection system at different reaction temperatures. Temperatures are shown at the top of each test strip. (**B**) Results of RPA-LFD detection system at different reaction times. The reaction times are shown at the top of each strip. The positions of the quality control and test lines are shown on the right side of the test strip. The color depth of the bands reflects the RPA reaction product content. The NC strips are template-free controls.

In order to obtain optimal reaction conditions for the RPA-LFD detection system, three different temperature conditions (25, 37 and 42 °C) and four time gradients (10, 15, 20 and 25 min) were set. The results of the detection system at three different temperatures are shown in Figure 1A. The 25 °C group was designed to explore the possibility of conducting the RPA reaction at room temperature, but no obvious T line was observed in this group. On the other hand, both the 37 °C and 42 °C groups showed obvious T lines. Since 37 °C is close to the human body temperature, it was chosen as the optimal reaction temperature.

As shown in Figure 1B, positive results were obtained across all four time gradients (ranging from 10–25 min), indicating that the shortest reaction time could be as brief as 10 min. However, the T line in the 10 min group had a lighter color compared to the other groups. The intensity of the T lines in the 15, 20 and 25 min groups was relatively consistent. Therefore, considering both the success rate and the time efficiency, a reaction time of 15 min was determined to be the optimal choice.

### 3.4. Specificity of RPA-LFD Detection System

**Figure 2:**
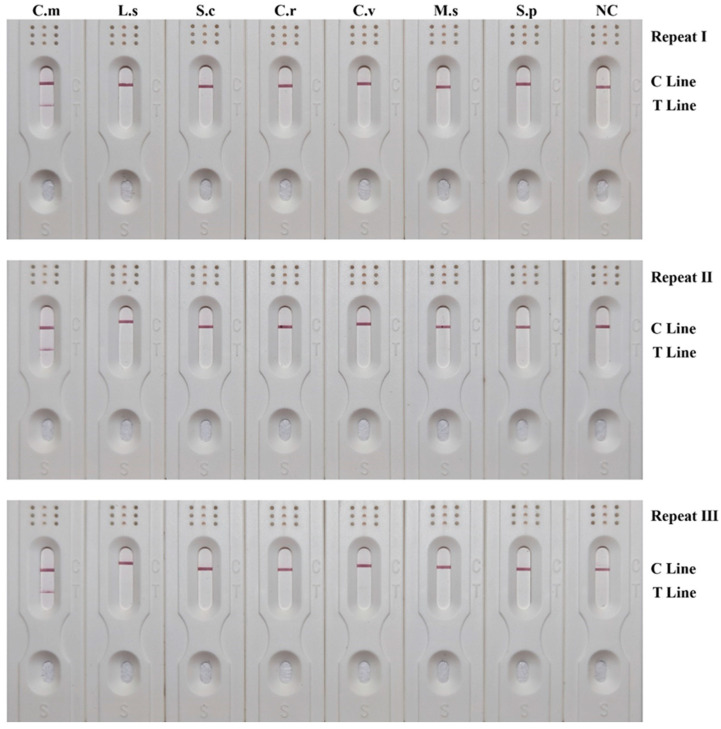
Specificity results of RPA-LFD detection system. The corresponding species name abbreviations were shown on the upper side, including *Chrysomya megacephala* (C.m), *Lucilia sericata* (L.s), *Sarcophaga crassipalpis* (S.c), *Chrysomya rufifacies* (C.r), *Calliphora vicina* (C.v), *Megaselia scalaris* (M.s) and *Sarcophaga peregrina* (S.p). The positions of the quality control and test lines are shown on the right side of the test strip. The color depth of the bands reflects the RPA reaction product content. The NC strips are template-free controls.

The results of the specificity assessment, as shown in Figure 2, suggested that only the test strips for *C. megacephala* exhibited red coloration in both the T lines and the C lines, indicative of positive results. In contrast, the test strips for the other necrophagous fly species showed no red coloration in the T lines, indicative of negative results. These findings were consistent across all three replications. These results indicated that the developed RPA-LFD detection system was species-specific, and could effectively differentiate *C. megacephala* from six other common necrophagous fly species.

### 3.5. Sensitivity of RPA-LFD Detection System

**Figure 3:**
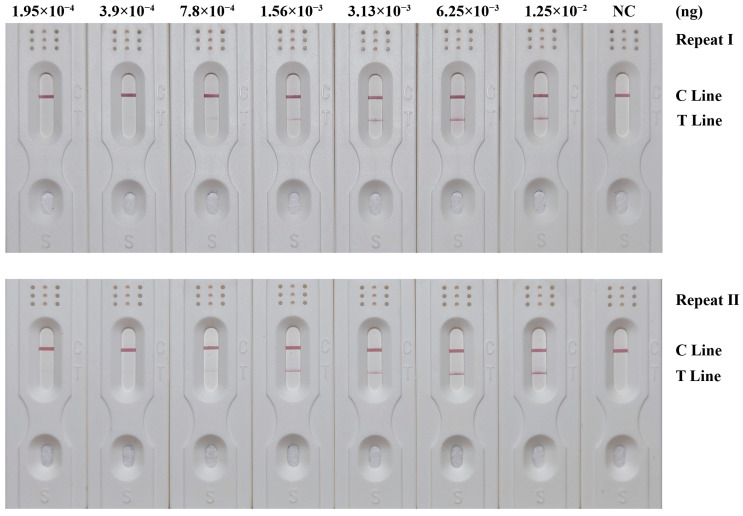
Sensitivity results of the RPA-LFD detection system. Synthetic target DNA fragment inputs were shown on the upper side, and dilutions were 2-fold. The positions of the quality control and test lines are shown on the right side of the test strip. The color depth of the bands reflects the RPA reaction product content. The NC strips are template-free controls.

The sensitivity of the RPA-LFD detection system was assessed using the synthetic target DNA fragments of *Cytb* of *C. megacephala*. The results are shown in Figure 3 and Table 5. Test strips with inputs below 7.8 × 10^−4^ ng of target DNA fragments did not exhibit any red coloration in the T lines. However, as the inputs exceeded 7.8 × 10^−4^ ng, a visible red coloration in the T lines emerged and intensified with higher inputs. Consistent results were obtained in both replication runs. Therefore, the sensitivity of the RPA-LFD detection system was determined to be 7.8 × 10^−4^ ng.

### 3.6. Effectiveness of RPA-LFD Detection System

**Figure 4:**
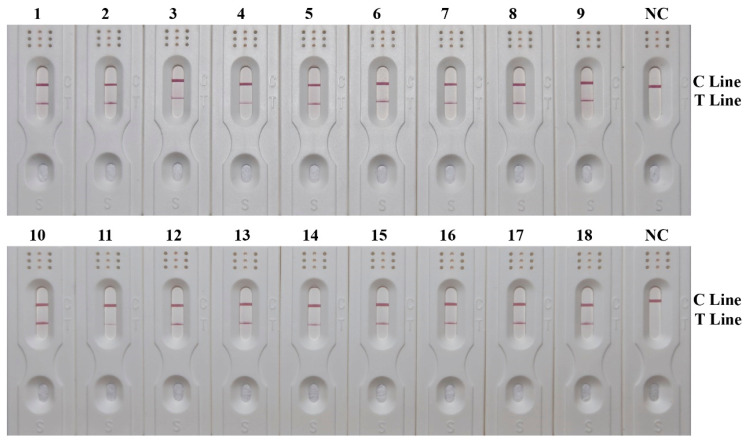
Effectiveness results of RPA-LFD detection system. The positions of the quality control and test lines are shown on the right side of the test strip. The color depth of the bands reflects the RPA reaction product content. The NC strips are template-free controls.

A total of 18 *C. megacephala* DNA samples from actual cases were used for RPA-LFD detection, with the results shown in Figure 4. All samples of *C. megacephala* were accurately identified, aligning with the sequencing results. The positive detection rate reached 100%, indicating that the developed RPA-LFD detection system could realize accurate and rapid detection of *C. megacephala*.

### 3.7. RPA-LFD Detection System in Combination with Rapid DNA Extraction Methods

**Figure 5:**
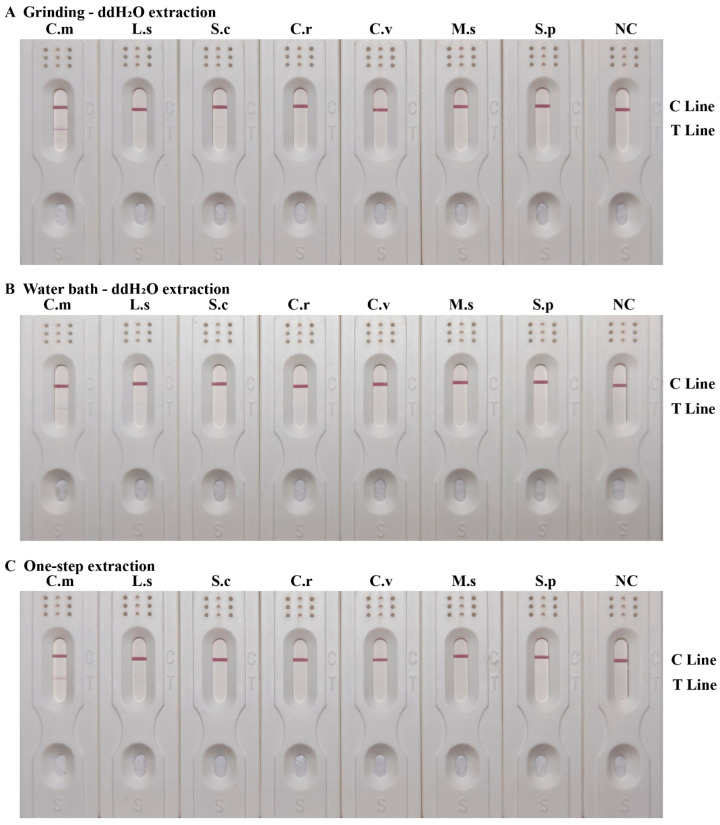
RPA-LFD detection system combined with rapid DNA extraction. (**A**) Grinding ddH_2_O extraction method. (**B**) Water bath ddH_2_O extraction method. (**C**) One-step extraction method. The corresponding species name abbreviations were shown on the upper side, including *Chrysomya megacephala* (C.m), *Lucilia sericata* (L.s), *Sarcophaga crassipalpis* (S.c), *Chrysomya rufifacies* (C.r), *Calliphora vicina* (C.v), *Megaselia scalaris* (M.s) and *Sarcophaga peregrina* (S.p). The positions of the quality control and test lines are shown on the right side of the test strip. The color depth of the bands reflects the RPA reaction product content. The NC strips are template-free controls.

In this study, we conducted a comparison of three rapid extraction methods in combination with the RPA-LFD detection system, as shown in Figure 5. The test strips of *C. megacephala* from all three groups exhibited red coloration in both the T lines and the C lines, indicative of positive results. The test strips of other necrophagous fly species from all three groups did not show any red coloration in the T lines, indicative of negative results. These results indicated that the RPA-LFD detection system, when used in combination with the three rapid DNA extraction methods, had good specificity.

When comparing the positive results among the three groups of *C. megacephala*, the red coloration in the T line obtained from the grinding ddH_2_O extraction method was comparable to that obtained from the one-step extraction method and was darker than that obtained from the water bath ddH_2_O extraction method. These results suggested that the performance of the grinding ddH_2_O extraction method was comparable to that of the one-step extraction method, which was less costly and time-consuming, and therefore had great potential for on-site detection when combined with the RPA-LFD detection system.

## 4. Discussion

In this study, an RPA-LFD detection system was successfully established based on the differential region of *Cytb* for the accurate and rapid identification of *C. megacephala*. The detection system had a minimum detection time of only 15 min at 37 °C, a sensitivity as low as 7.8 × 10^−4^ ng, a good specificity, and a 100% detection rate of *C. megacephala.* When combined with rapid DNA extraction methods, this detection system also had high practical value.

To date, several studies have used various molecular biology methods to identify necrophagous fly species, primarily including Sanger sequencing of DNA barcode, species-specific single nucleotide polymorphism (SNP)-based quantitative real-time PCR (q-PCR) and SNaPshot, etc. [38,39,40,41]. Harvey et al. (2003) used Polymerase Chain Reaction (PCR) in combination with Sanger sequencing to detect *COI* of *C. megacephala*, requiring a recommended template amount of 100 ng [38]; and GilArriortua et al. (2013) detected *Cytb* of *C. megacephala* with a minimum template amount of 10 ng [39]; and Jang et al. (2019) employed q-PCR to detect *C. megacephala*-specific SNP, requiring a minimum template amount of 1 ng [40]. The RPA-LFD detection system established in this study exhibited greater sensitivity and was superior to these commonly used molecular biology methods in detecting even mutilated insect samples. At the same time, the RPA-LFD system developed in this study was more time-efficient than commonly used molecular biology detection methods, providing reliable results in just 15 min with straightforward result interpretation. In contrast, Sanger sequencing and SNaPshot typically took several hours to complete, and even q-PCR required at least 70 min, accompanied by complex result analysis [40]. This study also explored the performance of the RPA-LFD detection system in combination with rapid DNA extraction methods, and found that DNA extraction from ground insect samples using ddH_2_O provided satisfactory results. The entire process, from insect DNA extraction to result visualization, took only 20 min, eliminating the need for specialized laboratory reagents and large-scale experimental instruments. Compared to these commonly used molecular methods for identifying necrophagous fly species, this innovative RPA-LFD detection system outperforms in speed, sensitivity, and convenience.

In addition, isothermal amplification techniques, such as loop-mediated isothermal amplification (LAMP) and RPA, are emerging as valuable molecular methods for insect species identification due to their simplicity, speed, and high specificity [42]. These methods are becoming increasingly popular among researchers, as they allow PCR reactions to be completed at a constant temperature in a short period without the need for large laboratory instruments. The published study indicated that the sensitivity of LAMP and RPA was significantly higher than that of traditional PCR [43]. However, the primer design for LAMP is more complex and challenging compared to RPA. Consequently, the RPA is simpler, faster, and may be more suitable for identifying necrophagous fly species in crime scenes. To date, there has been no research on the application of isothermal amplification techniques in forensic entomology, and our study has established a rapid and accurate detection system for *C. megacephala* based on RPA for the first time.

Despite its advantages, the application of the established RPA-LFD detection system may encounter several challenges. First, in this study, the species-specific primers were designed based on common necrophagous fly species collected from actual forensic cases and validated using the Primer-BLAST on the NCBI website. However, not all *calliphorids* that typically colonize remains are included, and some closely related non-target species lack *Cytb* sequences in the NCBI database, and the gene flow is not considered, which may lead to potential cross-reactivity. In the future, we will expand the scope of specificity validation for the established RPA-LFD detection system to further ensure it is species-specific. Second, there may be potential contamination risks during DNA extraction and RPA reaction. While crime scenes are often well-ventilated, DNA extraction or RPA reaction is generally performed in a single EP or PCR tube, and we recommend running negative control samples during the process, which will most likely further reduce the risk of potential contamination. Third, the diverse environments of crime scenes necessitate that the established RPA-LFD detection system show stability to maximize its practical value. Our research team has previously demonstrated that the detection system based on RPA and LFD technologies is reproducible across different times, kit lots, investigators, and experimental conditions [44]. In the future, we will apply the established RPA-LFD detection system to various crime scenes to further confirm its effectiveness. Finally, while the established RPA-LFD detection system is designed for a known necrophagous fly species and is simple, rapid, and accurate, it may require multiple RPA-LFD systems when dealing with unknown species. This could significantly increase costs and the time required for analysis. Considering these factors, the RPA-LFD detection system established in our study is particularly suitable for forensic grassroots personnel to quickly determine the true species of *C. megacephala* in the field or in remote areas, thus replacing the standard DNA extraction, PCR and sequencing of *COI* that rely on specialized expertise and laboratory facilities.

The selection of target genes was crucial for achieving accurate insect species identification. In this study, we compared the *Cytb* sequences of case-related necrophagous fly species, and designed species-specific RPA primer and probe. The commonly used DNA “barcodes” for identifying necrophagous fly species included *Cytb*, *COI* [38,45,46] and internal transcribed spacer 2 (ITS2) [47]. However, when comparing the sequences of the *COI* and ITS2 regions of case-related necrophagous fly species, minimal differences were found between these species, which made it difficult to design species-specific RPA primers. The study published by GilArriortua et al. (2013) compared *Cytb* of six necrophagous fly species, and found that *Cytb* had more variable positions [39]. Meanwhile, there have been studies in the field of rapid detection using *Cytb* as a target for RPA to identify vertebrates (chicken and duck) [48,49]. Building on this, this study attempted to use *Cytb* as a target for species-specific RPA primer and probe design. By comparing the *Cytb* sequences of seven common necrophagous fly species, we identified significant differences and successfully validated the feasibility of *Cytb* as a target for RPA in the detection of arthropods for the first time.

The RPA is a class of point-of-care testing (POCT) due to its simplicity, responsiveness, and lack of reliance on large-scale instruments. Downstream of the RPA reaction, various methods can also be combined to visualize the detection results, such as LFD [50], real-time fluorescence [51,52], and gel electrophoresis [53]. The aim of this study was to establish a rapid and accurate detection method suitable for field environments, thus the LFD combined with visualization by the naked eye was chosen as the downstream approach following the RPA reaction. Finally, a rapid and accurate RPA-LFD detection system for *C. megacephala* was successfully established. This system addresses the gap in the field of rapid species identification for necrophagous flies and is superior to the commonly used necrophagous fly species identification methods in terms of cost, speed, sensitivity and convenience, which fully proves that the RPA-LFD detection system has a broad application prospect in the field of forensic science. At present, there are commercially available multi-detection test strips that enable simultaneous detection of multiple target proteins or genes on a single strip, such as Quick Stix Combo Kit of Envirologix and HybriDetect Nucleic Acid Strip Kit of Milenia. Our future research will be based on the existing single-species RPA-LFD detection system and further explore and establish a multi-species RPA-LFD detection system for necrophagous flies.

## 5. Conclusions

In this study, we innovatively constructed a rapid and accurate detection system for the identification of *C. megacephala* based on *Cytb* using a combination of RPA and LFD technologies. The RPA-LFD detection system was established to amplify in just 15 min at 37 °C, with a sensitivity of 7.8 × 10^−4^ ng, a good species specificity, and a positive detection rate of up to 100%. When the RPA-LFD detection system was combined with the grinding ddH_2_O extraction method (a rapid DNA extraction method), the species specificity was still very good, and the process, from species acquisition to visualization of detection results, could be completed in less than 20 min. In conclusion, this innovative RPA-LFD detection system is rapid and accurate in identifying *C. megacephala*, and it provides an ideal solution for species identification in environments with limited access to laboratory equipment.

## Figures and Tables

**Table 1:** Primer for *COI* in necrophagous flies.

Primer	Sequence (5′–3′)	Primer Length (bp)	Amplification Length (bp)
Barcode-658	F: GGTCAACAAATCATAAAGATATTGG	25	658
	R: RAAACTTCAGGRTGACCAAAGAATCA	26	

**Table 4:** Primers and probe for recombinase polymerase amplification (RPA) of *C. megacephala*.

Assay	Primer	Sequence (5′–3′)	Primer Length (nt)	Amplification Length (bp)
Basic RPA	CM-RPA-1	F: TTTACCATTTATTGTTCTAGCTGCAACTCTT	31	256
		R: TTGAATATGAACTGGAGTAACTAAAGGATT	30	
	CM-RPA-2	F: TACCATTTATTGTTCTAGCTGCACGTCGT	29	254
		R: TTGAATATGAACTGGAGTAACTAAAGGATT	30	
	CM-RPA-3	F: TTTTATCCCAGCCAATCCTTTAGTTACTCC	30	199
		R: ATTAATAGGGTAGAATTGAATACCTCGGAAC	31	
RPA-LFD	CM-RPA-2	mF: [FAM]-TACCATTTATTGTTCTAGCTGCACGTCGT	29	254
		mR: [Biotin]-TTGAATATGAACTGGAGTAACTAAAGGATT	30	
	Probe	[FAM]-ATTTTATTAGTATTAATTAATCCTTACTTAC [TNF]TTGGTGACCCTGATAAT-[C3 spacer]	48	

**Table 5:** Sensitivity of the RPA-LFD detection system.

Repeat	Dilution Factor						
	2^0^ (1.25 × 10^−2^ ng)	2^1^ (6.25 × 10^−3^ ng)	2^2^ (3.13 × 10^−3^ ng)	2^3^ (1.56 × 10^−3^ ng)	2^4^ (7.8 × 10^−4^ ng)	2^5^ (3.9 × 10^−4^ ng)	2^6^ (1.95 × 10^−4^ ng)
I	+++	+++	++	++	+	**−**	**−**
II	+++	+++	++	++	+	**−**	**−**

**Table 2:** Necrophagous insects in 45 real cases in south and north China (Changsha and Beijing) during 2018–2023.

Case	Sample	Species Identified by Sequencing
S2018-18		*Chrysomya pinguis* (Walker)
S2018-168		*Chrysomya megacephala*
S2018-219	1	*Synthesiomyia nudiseta* (van der Wulp)
	2	*Chrysomya rufifacies*
S2020-107		*Sarcophaga peregrina*
S2022-18	1–3	*Sarcophaga peregrina*
S2022-34	1, 2	*Aldrichina grahami* (Aldrich)
S2022-108	1–3	*Chrysomya megacephala*
S2022-110	1	*Lucilia sericata*
	2, 3	*Chrysomya megacephala*
S2022-113	1	*Creophilus maxillosus* (Linnaeus)
	2, 7	*Chrysomya megacephala*
	3	*Megaselia scalaris*
	4	*Dohrniphora cornuta* (Bigot)
	5, 6	*Muscina stabulans* (Fallén)
S2022-139	1–3	*Chrysomya megacephala*
S2022-150	1–4	*Chrysomya megacephala*
S2023-35	1	*Chrysomya rufifacies*
S2023-107	1, 2, 4–9	*Chrysomya pinguis*
	3	*Chrysomya rufifacies*
S2023-159		*Chrysomya megacephala*
S2023-161		*Sarcophaga peregrina*
S2023-202	1, 4	*Sarcophaga peregrina*
	2	*Chrysomya megacephala*
	3	*Musca domestica* (Linnaeus)
N1	1–5	*Sarcophaga crassipalpis*
N2		*Lucilia sericata*
N3		*Sarcophaga crassipalpis*
N4		*Sarcophaga portschinskyi* (Rohdendorf)
		*Sarcophaga peregrina*
N5		*Sarcophaga crassipalpis*
N6		*Megaselia scalaris*
N7		*Sarcophaga crassipalpis*
		*Chrysomya rufifacies*
N8		*Sarcophaga crassipalpis*
		*Lucilia sericata*
N9		*Sarcophaga crassipalpis*
N10	1–3	*Calliphora vicina*
N11	1, 2	*Calliphora vicina*
	3	*Lucilia sericata*
N12	1–3	*Calliphora vicina*
N13		*Calliphora vicina*
N14	1, 2	*Calliphora vomitoria* (Linnaeus)
	3, 4	*Triceratopyga calliphoroides* (Rohdendorf)
	6	*Dermestes coarctatus* (Harold)
N15	2	*Lucilia sericata*
	3	*Sarcophaga portschinskyi*
N16	1–3	*Lucilia ampullacea* (Villeneuve)
N17	1	*Lucilia sericata*
	2, 3	*Chrysomya megacephala*
N18	1, 2	*Lucilia sericata*
	3	*Sarcophaga dux* (Thomson)
N19	1, 2	*Megaselia scalaris*
	3	*Blattella germanica* (Linnaeus)
N20	1, 2	*Sarcophaga crassipalpis*
	3	*Lucilia sericata*
N21	1–3	*Lucilia sericata*
N22	1–3	*Megaselia scalaris*
N23	1, 2	*Chrysomya megacephala*
	3	*Chrysomya rufifacies*
N24	1	*Chrysomya megacephala*
	2	*Sarcophaga dux*
	3, 4	*Chrysomya rufifacies*
N25	1	*Megaselia scalaris*
	2	*Synthesiomyia nudiseta*
	3	*Sarcophaga crassipalpis*
	4	*Sarcophaga nathani* (Lopes)
N26	1	*Blattella germanica*
	3–4	*Sarcophaga portschinskyi*
N27	1–4	*Lucilia sericata*
N28	1–3	*Calliphora vicina*
N29	1, 4	*Calliphora nigribarbis* (Vollenhoven)
	3	*Lucilia sericata*
	5, 6	*Protophormia terraenovae* (Robineau-Desvoidy)

**Table 3:** Data analysis of necrophagous insects in real cases in south and north China.

Species Name	Total Cases	Cases from South China	Cases from North China
*Chrysomya megacephala*	11	8	3
*Lucilia sericata*	11	1	10
*Sarcophaga crassipalpis*	8		8
*Chrysomya rufifacies*	6	2	4
*Calliphora vicina*	5		5
*Megaselia scalaris*	5	1	4
*Sarcophaga peregrina*	5	4	1
*Sarcophaga portschinskyi*	3		3
*Chrysomya pinguis*	2	2	
*Blattella germanica*	2		2
*Sarcophaga dux*	2		2
*Sarcophaga nathani*	2		2
*Calliphora nigribarbis*	1		1
*Protophormia terraenovae*	1		1
*Muscina stabulans*	1	1	
*Triceratopyga calliphoroides*	1		1
*Creophilus maxillosus*	1	1	
*Calliphora vomitoria*	1		1
*Lucilia ampullaceal*	1		1
*Musca domestica*	1	1	
*Dohrniphora cornuta*	1	1	
*Aldrichina grahami*	1	1	
*Synthesiomyia nudiseta*	1	1	
*Dermestes coarctatus*	1	1	

## Data Availability

The data presented in this study are contained in the manuscript and available on request from the corresponding author.

## Supplementary Materials

The following supporting information can be downloaded at: https://www.mdpi.com/article/10.3390/insects15121008/s1, Figure S1: RPA Primers screening results. The amplified bands targeting *Cytb* were displayed. The corresponding species name abbreviations were shown on the upper side, including *Chrysomya megacephala* (C.m), *Lucilia sericata* (L.s), *Sarcophaga crassipalpis* (S.c), *Chrysomya rufifacies* (C.r), *Calliphora vicina* (C.v), *Megaselia scalaris* (M.s) and *Sarcophaga peregrina* (S.p). The names of the three sets of primers were shown on the lower side. The DNA ladder was shown on the left side, indicating the band sizes.
