# Supplementary material for: Rapid and Accurate Detection of Chrysomya megacephala (Diptera: Calliphoridae) Using Recombinase Polymerase Amplification Combined with Lateral Flow Dipstick
Source: Insects. 2024 Dec 20;15(12):1008. doi: 10.3390/insects15121008 (PMC11677130; doi:10.3390/insects15121008)
Supplement: Supplementary file 1 [file insects-15-01008-s001.zip › Figure S1.pdf]

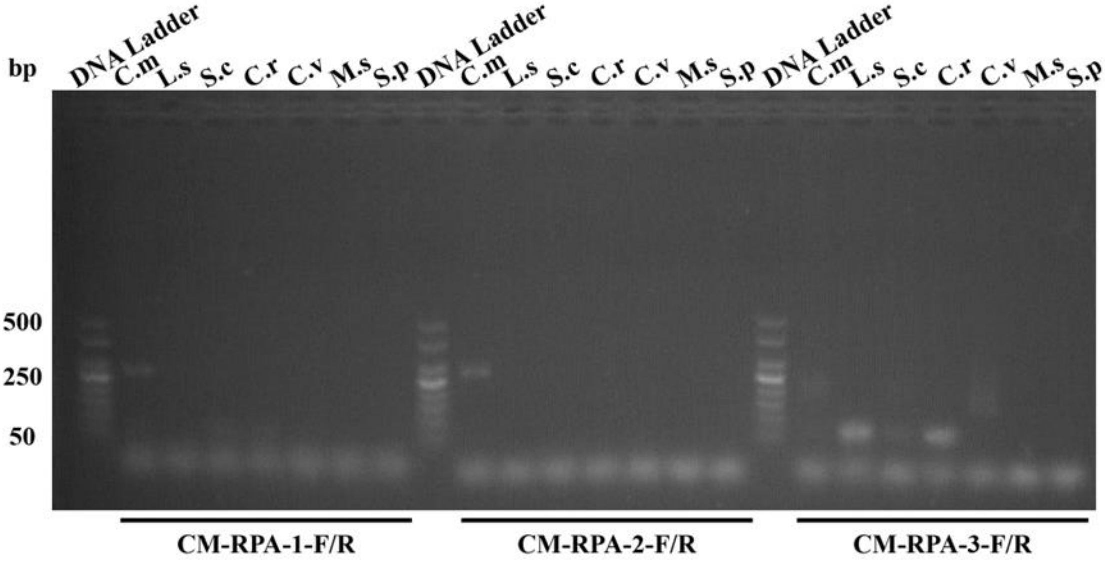

Figure S1. RPA Primers screening results. The amplified bands targeting *Cytb* were displayed. The corresponding species name abbreviations were shown on the upper side, including *Chrysomya megacephala* (C.m), *Lucilia sericata* (L.s), *Sarcophaga crassipalpis* (S.c), *Chrysomya rufifacies* (C.r), *Calliphora vicina* (C.v), *Megaselia scalaris* (M.s) and *Sarcophaga peregrina* (S.p). The names of the three sets of primers were shown on the lower side. The DNA ladder was shown on the left side, indicating the band sizes.
